# Regulation of the NRG1/ErbB4 Pathway in the Intrinsic Cardiac Nervous System Is a Potential Treatment for Atrial Fibrillation

**DOI:** 10.3389/fphys.2018.01082

**Published:** 2018-08-21

**Authors:** Xiaoya Zhou, Zhuo Wang, Bing Huang, Shenxu Yuan, Xia Sheng, Lilei Yu, Guannan Meng, Yuhong Wang, Sunny S. Po, Hong Jiang

**Affiliations:** ^1^Department of Cardiology, Renmin Hospital of Wuhan University, Wuhan, China; ^2^Sir Run Run Shaw Institution of Clinical Medicine and Department of Cardiology, Sir Run Run Shaw Hospital Affiliated to Medical College of Zhejiang University, Hangzhou, China; ^3^Heart Rhythm Institute, University of Oklahoma Health Sciences Center, Oklahoma City, OK, United States

**Keywords:** neuregulin-1, ErbB4, atrial fibrillation, autonomic nervous system, ganglionated plexi

## Abstract

**Background:** The NRG1/ErbB4 signaling mechanism has been widely studied in the central nervous system for many years. However, the role of this pathway in modulating the intrinsic cardiac nervous system is largely unknown.

**Objective:** The present study investigated whether the NRG1/ErbB4 signaling system affects the activity of major atrial ganglionated plexi (GP) in a paroxysmal atrial fibrillation (AF) model by 6-h rapid atrial pacing (RAP).

**Methods:** Twenty-four dogs were randomly divided into (1) a control group (saline microinjections into GP), (2) RAP group (saline microinjections into GP plus 6 h-RAP), (3) NRG1 group (microinjections of neuregulin-1 into GP plus 6 h-RAP) and (4) NRG1 + ERA group (microinjections of neuregulin-1 and ErbB4 receptor antagonist-ERA into GP plus 6 h-RAP). The effective refractory period (ERP), window of vulnerability (WOV), anterior right GP (ARGP) function and neural activity were measured. ARGP tissues were excised for histological study and western blotting.

**Results:** When compared to the control group, 6 h-RAP produced a significant (1) decrease in ERP, an increase in ΣWOV, (2) an increase in ARGP neural activity and neural function, and (3) an increase in c-fos and nerve growth factor protein expression in the ARGP. However, microinjection of NRG1 into the ARGP prior to RAP prevented ERP shortening and AGRP activity enhancement and inhibited the expression of c-Fos and NGF proteins. Furthermore, these changes were significantly attenuated by pretreatment with an ErbB4 receptor antagonist.

**Conclusion:** The NRG1/ErbB4 signaling pathway may exist in the GP, and activation of this pathway suppressed RAP-induced GP activation, atrial electrical remodeling and AF.

## Introduction

The extrinsic and intrinsic autonomic nervous systems control the heart. The intrinsic autonomic nervous system consists of multiple ganglionated plexi (GP), which are called the heart’s ‘little brain’ (Wake and Brack, 2016). Numerous preclinical studies have revealed that hyperactivity of the major atrial GP plays a crucial role in the initiation and maintenance of atrial fibrillation (AF) (Benjamin et al., 1998; Lu et al., 2008; Tan et al., 2008; Park et al., 2012). Interventions that inhibit GP neural activity or directly injure the GP neurons suppress AF in animal models and patients (Lu et al., 2008; Katritsis et al., 2013; Yu et al., 2013; Stavrakis et al., 2015; Lo et al., 2016). However, the mechanism(s) underlying GP inhibition are not clear.

Neuregulins (NRGs) play an important role in neural development and brain activity homeostasis. NRG-1 is distributed widely in the central and peripheral nervous systems (Buonanno and Fischbach, 2001; Thompson et al., 2007). ErbB4 is the only known receptor that exhibits high affinity for NRG1 and an active tyrosine kinase domain (Tan et al., 2011). NRG1/ErbB4 signaling was implicated in neural development, including circuitry generation, axon ensheathment, neurotransmission, and synaptic plasticity (Buonanno and Fischbach, 2001). The signaling pathway has also been shown to regulate neuronal excitability and synaptic plasticity in the adult brain (Corfas et al., 2004; Mei and Nave, 2014).

The NRG1/ErbB4 signaling networks function widely in the central nervous system, and NRG1 suppresses excitatory neuronal activity via activation of the ErbB4 receptor (Fazzari et al., 2010; Tan et al., 2011). A previous study found a significant association between NRG-1 levels and the presence of paroxysmal AF (Shao et al., 2014). We hypothesized that an NRG1/ErbB4 signaling pathway exists in the cardiac nervous system (GP), and activation of this signaling pathway influences AF initiation and maintenance.

## Materials and Methods

All experimental protocols conformed to the Guideline for the Care and Use of Laboratory Animals published by the United States National Institutes of Health (NIH Publication, revised 1996), and the Committee on the Ethics of Animal Experiments of Wuhan University approved all protocols.

### Animal Preparation

Twenty-four canines weighing 20–25 kg were included in this study. All surgeries were performed under anesthesia with 3% sodium pentobarbital with an initial dose of 1 ml/kg (IV) and a maintenance dose of 2 ml/h (IV). Body surface electrocardiography and blood pressure were recorded using a computer-based laboratory system (Lead7000, Jinjiang Inc., Chengdu, China). The core body temperature of the dogs was maintained at 36.5°C ± 1.5°C. Bilateral thoracotomy was performed at the fourth intercostal space to expose the bilateral atria, atrial appendages, pulmonary veins and atrial GP. Multielectrode catheters were sutured at the following seven sites: right superior pulmonary vein (RSPV), right inferior pulmonary vein (RIPV), right atrium (RA), right atrial appendage (RAA), left superior pulmonary vein (LSPV), left inferior pulmonary vein (LIPV), and left atrium (LA). High frequency stimulation (20 Hz, 0.1-ms pulse duration) was applied to identify the location of the major GPs.

### Programmed Stimulation

Six hours of RAP (20 Hz, 2× threshold) was delivered at the left atrial appendage to induce acute atrial remodeling (Sheng et al., 2011). RAP was temporarily stopped for 5–10 min to measure the effective refractory period (ERP). ERP was determined using a programmed stimulation that consisted of eight consecutive stimuli (S1–S1 interval = 330 ms) followed by a premature stimulus (S1–S2 interval). The S1–S2 intervals were decreased from 150 ms initially in decrements of 10 ms, then 2 ms when approaching ERP. We used the window of vulnerability (WOV) as a quantitative measure of AF inducibility. The interval between the longest and shortest S1–S2 interval (in ms) at which AF was induced was defined as the WOV. The cumulative WOV (ΣWOV) was the sum of the WOVs at all sites in each dog.

### Measurement of Anterior Right GP (ARGP) Function

Four major GPs were located anatomically. The anterior right GP (ARGP) was located at the right superior PV – atrial junction; the inferior right GP (IRGP) was situated at the junction of inferior vein cava and both atria; the superior left GP (SLGP) was located near the left superior PV – atrial junction and left pulmonary artery; and the inferior left GP (ILGP) was located at the left inferior PV – atrial junction. The ARGP was precisely localized via application of high-frequency stimulation (20 Hz, square wave pulse, 0.1 ms duration, 2–5 V) using a Grass stimulator (model S88, Grass Instruments, Quincy, MA, United States). ARGP function was measured as the maximal change in sinus rate (SR) in response to direct electrical stimulation of ARGP (Sha et al., 2011; Wang et al., 2016). We used four incremental voltage levels to stimulate the ARGP (level 1: 1–4 volts; level 2: 5–7.5 volts; level 3: 8–10 volts; level 4: 10–15 volts). Each high-frequency stimulation lasted ≤30 s, and we waited until the SR returned to baseline before delivering the next round of stimulation. Data are presented as the percent of maximal SR slowing (compared to the baseline state) induced by ARGP stimulation at different levels of stimulation voltages.

### Measurement of GP Neural Activity

A coated tungsten microelectrode (2 cm in length) with an exposed tip of 50 μm was inserted into the fat pad containing the ARGP, and an impedance of 9–12 MΩ at 1,000 Hz was mounted on a micromanipulator. Electrical signals generated by the ARGP were recorded using a PowerLab data acquisition system (8/35, ADInstruments, Bella Vista, New South Wales, Australia), and the signals were amplified using an amplifier (DP-304, Warner Instruments, Hamden, CT, United States) with bandpass filters set at 300 Hz to 1 kHz and an amplification range of 30–50 times (Yu et al., 2013). Neural activity was characterized using the recorded amplitude and frequency that was used to quantitate neural activity. Neural activity was defined as deflections with a signal-to-noise ratio greater than 3:1 (Yu et al., 2014).

### Experimental Protocol

**FIGURE 1:**
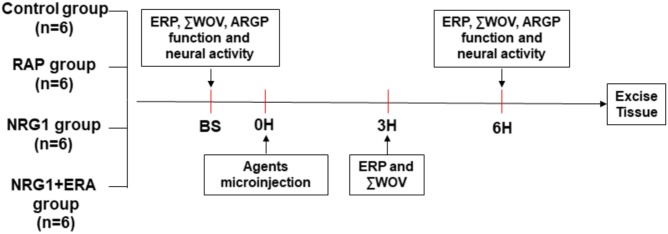
Flow diagram of the present study. RAP was performed after saline, NRG1 or NRG1 plus ERA microinjection into four atrial GP and LOM, except in the control group. ERP and ΣWOV at atrial and pulmonary vein sites were examined at baseline, the end of 3rd hour and the end of the 6th hour. ARGP function and neural activity were examined at baseline and the end of 6th hour, respectively. GP tissues were excised for histological studies at the end of the 6th hour.

Twenty-four dogs were randomly divided into the control group, RAP group, NRG1 group, and NRG1 plus the ErbB4 receptor antagonist ERA (NRG1 + ERA) group (**Figure 1**). Saline was microinjected into the four major GP (ARGP, inferior right GP, superior left GP and inferior left GP) and the ligament of Marshall (LOM) followed by 6 h of sham RAP in the control group. Saline was microinjected into the four major GPs and the LOM followed by 6 h of RAP in the RAP group. NRG1 (10 μM) was microinjected into the four major GP and the LOM followed by 6 h of RAP in the NRG1 group. NRG1 (10 μM) and ERA (PD158780, an ErbB4 receptor antagonist, 2 mM) were co-injected into the four major GP and the LOM followed by 6 h of RAP in the NRG1 + ERA group. All agents were administered into the GP and LOM location using a 34 G stainless steel needle connected to a micropump (Longer Precision Pump TJ-4A, Hebei, China) at a rate of 0.5 μl/min in a total volume of 5–10 μl. Atrial ERP and WOV were determined at baseline and the end of the 3rd and 6th hours of RAP. ARGP function and ARGP neural activity were measured at baseline and the end of the 6th hour of RAP (**Figure 1**).

### Western Blot Analysis and Histological Studies

Anterior right GP was excised and washed with saline at the end of the experiment. Tissue for western blot analysis was dissected into small portions and maintained at -80°C for subsequent analyses. Tissue for histological studies was fixed with 4% paraformaldehyde. The protein expression levels of c-fos and nerve growth factor (NGF) in ARGP were analyzed using western blot analysis. The primary antibodies used were anti-c-fos (Abcam, Cambridge, England) and anti-NGF (Santa Cruz, Dallas, TX, United States). The membrane was washed and incubated with secondary anti-rabbit antibodies at 37°C for 2 h. Antibody-binding protein bands were visualized and quantified. Immunofluorescence staining was used to confirm the expression and location of ErbB4 in atrial GP. Paraffin-embedded GP tissues were cut transversely into 5-μm sections. The primary antibodies used were anti-ErbB4 (Santa Cruz, Dallas, TX, United States) and anti-parvalbumin (Abcam, Cambridge, England). Quantitative analyses were performed using commercially available software (Image Pro Plus, Media Cybernetics, Inc., Rockville, MD, United States).

### Statistical Analysis

Continuous variables are presented as the means ± standard deviations (SD). A one-way analysis of variance (ANOVA) followed by Tukey’s test was used for *post hoc* multiple comparisons of c-fos, NGF, ErbB4, and parvalbumin levels between different groups. A two-way ANOVA followed by Tukey’s test was used to examine differences in ERP, ΣWOV, ARGP function and ARGP neural activity. SPS22.0 (SPSS Inc., Chicago, IL, United States) was used for data analysis and graphing, and two-tailed *P* < 0.05 was considered significant.

## Results

### ERP and ΣWOV

**FIGURE 2:**
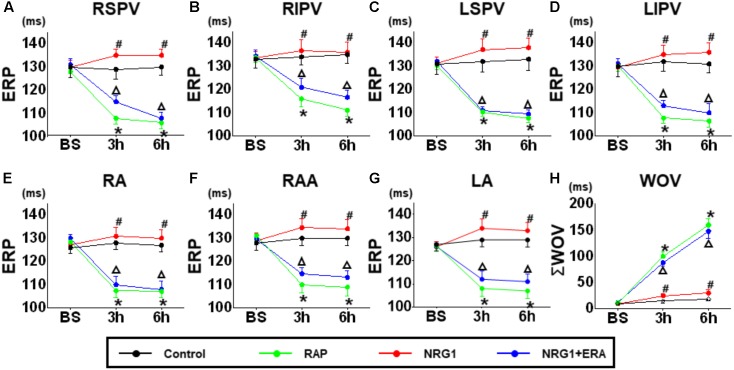
Changes in ERP and ΣWOV. **(A–G)** The ERP changes at different time points in the control group, RAP group, NRG1 group and NRG1 + ERA group. **(H)** The ΣWOV changes at different time points in the four groups. The ERP was shortened, and ΣWOV was widened in atrial and pulmonary vein sites in the RAP group. NRG1 alleviated the effect of RAP, and ERA blocked the beneficial effects of NRG1 on ERP and ΣWOV. ^∗^*p* < 0.05 vs. NRG1 group. ^#^*p* < 0.05 vs. RAP group. ^Δ^*P* < 0.05 vs. the NRG1 group.

No significant difference in ERP was demonstrated at any site among all groups. The 6 h-RAP treatment resulted in a significant decrease in the ERP during the first 3 h of RAP at all pulmonary vein and atrial recording sites (**Figure 2**). Microinjection of NRG1 prevented ERP shortening and ΣWOV increase. However, the effects of NRG1 were blocked in the NRG1 + ERA group. The ERP at all sites was significantly shortened, and the ΣWOV was significantly increased (**Figure 2**).

### GP Function

**FIGURE 3:**
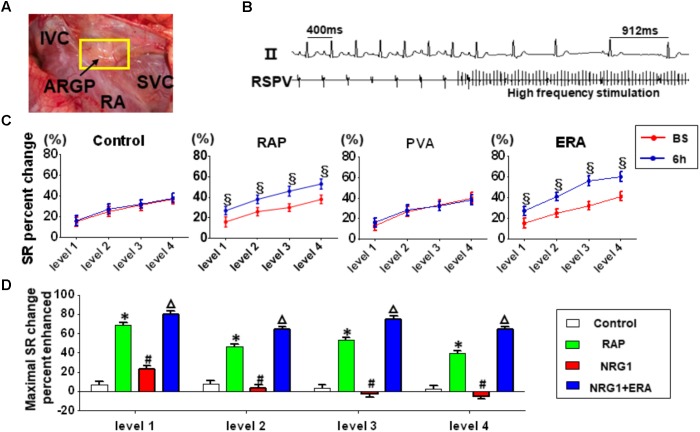
Changes in ARGP function. **(A)** The position of ARGP. **(B)** Representative example of the SR slowing response to ARGP stimulation. **(C)** ARGP function assessed using the SR slowing responses to ARGP stimulation before and after 6 h of RAP. **(D)** Percent of maximal SR change induced by different interventions after 6 h of RAP. ^§^ *P* < 0.05 vs. baseline; ^∗^*P* < 0.05 vs. the control group; ^#^*P* < 0.05 vs. the RAP group; ^Δ^*P* < 0.05 vs. the NRG1 group.

A significant slowing of SR was observed at baseline because of the high-frequency stimulation of the ARGP in all groups(**Figure 3**). The ability of ARGP stimulation to slow SR was significantly augmented after 6 h of RAP in the RAP and NRG1 + ERA groups (**Figure 3C**). **Figure 3D** illustrates that the SR slowing responses were augmented by 6 h of RAP at all levels of ARGP stimulation, but NRG-1 microinjection prevented these augmented responses. However, ERA and NRG1 co-injection produced similar effects as RAP alone.

### GP Neural Activity

**FIGURE 4:**
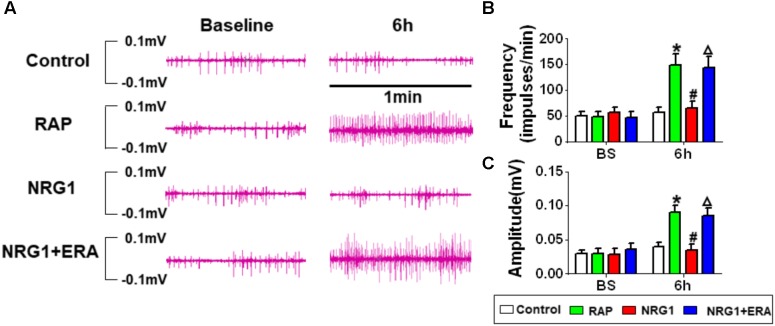
Changes in ARGP neural activity. **(A)** Representative ARGP neural activity in the control, RAP group, NRG1 group and NRG1 + ERA group. **(B)** Quantitative analysis of the frequency of ARGP neural spikes. **(C)** Quantitative analysis of the amplitude of ARGP neural spikes. ^∗^*P* < 0.05 vs. the control group; ^#^*P* < 0.05 vs. the RAP group; ^Δ^*P* < 0.05 vs. the NRG1 group.

**Figure 4A** shows the changes of ARGP neural activity in each group. There was no significant difference at baseline among all groups. No significant difference was found at different time points in the control group. In the RAP group, 6 h-RAP resulted in a significant increase in ARGP neural activity compared to baseline. However, **Figures 4A–C** shows that RAP-induced ARGP neural activation was prevented in the NRG1 group (both in frequency and amplitude). However, ARGP activity increased significantly in the NRG1 + ERA group compared to that in the NRG1 group.

### C-fos and NGF

**FIGURE 5:**
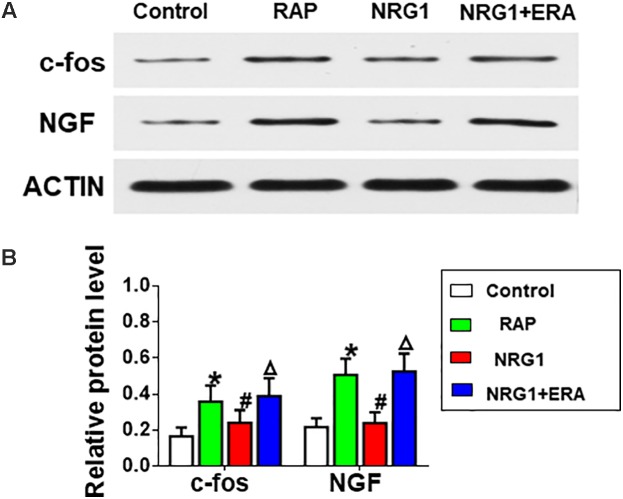
Changes in c-fos and NGF expression. **(A)** Representative examples of the protein levels of c-fos and NGF. **(B)** Quantitative analysis of the protein expression levels of c-fos and NGF. ^∗^*P* < 0.05 vs. the control group; ^#^*P* < 0.05 vs. the RAP group; ^Δ^*P* < 0.05 vs. the NRG1 group.

The protein levels of c-fos and NGF in the ARGP were much higher in the RAP group than in the control group. However, NRG1 microinjection significantly attenuated the RAP-induced expression of c-fos and NGF protein, and the ErbB4 receptor antagonist ERA prevented the inhibitory effects (**Figure 5**).

### Parvalbumin (PAV)-Positive Neurons and ErbB4 Receptor

**FIGURE 6:**
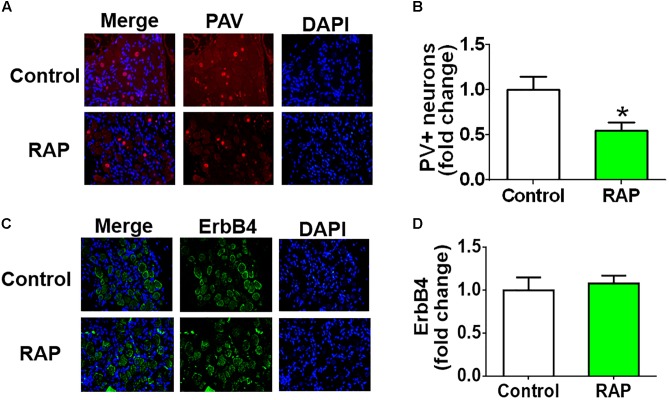
Parvalbumin (PAV) and ErbB4 staining before and after 6 h of RAP. **(A)** Representative example of PAV-immunoreactive neurons (40×, red) within the ARGP. **(B)** Quantitative analysis of the PAV-positive neurons. **(C)** Representative example of ErbB4-immunoreactive neurons (40×, green) within the ARGP. **(D)** Quantitative analysis of the ErbB4 positive cells. ^∗^*p* < 0.05 vs. control group.

Immunofluorescence staining of PAV and DAPI indicated the presence of PAV-positive neurons in atrial GP, and the expression of PAV-positive neurons decreased markedly after 6 h-RAP (**Figure 6**). In contrast, 6 h-RAP did not affect the high levels of ErbB4 expression.

## Discussion

### Major Finding

NRG1/ErbB4 signaling has been widely studied in the central nervous system for many years (Hu et al., 2014; Gonzalez-Burgos et al., 2015). To the best of our knowledge, this study is the first report of the NRG1/ErbB4 signaling pathway in the cardiac autonomic nervous system. Local microinjected NRG1 may inhibit the activity and function of ARGP and decrease AF inducibility. An ErbB4 receptor antagonist (PD158780) abolished the protective effect of NRG1, which indicates that NRG1 functions via the ErbB4 receptor-related signaling pathway.

### The Possible Mechanism of the NRG1/ErbB4 Signaling Pathway in AF

Most intrinsic nerve cell bodies reside in the fat pads on the epicardial surface, with axons that form connections with other nearby neurons, which create networks that appear as GP. The GP are connected with stellate ganglia in the thoracic and lumbar spine dorsal root ganglia (Kummer and Oberst, 1993; Tsai et al., 2017), which connect with the posterior and lateral hypothalamic nuclei and locus coeruleus in the brain stem (Jansen et al., 1995). The widespread distribution of these ganglia and the extensive networks of the plexuses suggest that intrinsic nerves play an important role in the initiation and maintenance of AF (Chen et al., 2014).

NRG1 is an expressed epidermal growth factor (EDF)-like protein, and it is essential for the normal development of the nervous system. NRG1 regulates target cell differentiation (Falls, 2003), neurotransmitter receptor expression (Buonanno and Fischbach, 2001), interneuronal peripheral nerve synapse development (Meyer and Birchmeier, 1995) and Schwann cell survival (Escher et al., 2005) in the peripheral nervous system. The present study found that microinjection of extraneous NRG1 inhibited the activity and function of ARGP and decreased AF inducibility in a 6 h-RAP-induced AF model in canines. We speculated that NRG-1 would exert protective effects in the maintenance of atrial electrophysiological stability. This hypothesis is consistent with a previous study indicating that exogenous NRG-1 administration exerted a neuroprotective effect in the central nervous system (Xu et al., 2005).

The activity of NRG1 is primarily attributed to the activation of ErbB receptors and induction of acetylcholine receptor expression (Falls, 2003; Mei and Xiong, 2008). Kuramochi et al. (2004) demonstrated that NRG-1 activated ErbB4 receptor phosphorylation in isolated cardiac myocytes. Our findings indicate that pretreatment with an ErbB4 receptor antagonist (PD158780) attenuated the protective effects of NRG1, which indicated that NRG1 inhibited GP neural hyperactivity via the ErbB4 receptor. We suggest that the ErbB4 receptor also exists in the cardiac autonomic nervous system, and NRG1 activation improves atrial electrophysiological stability via an ErbB4 receptor-related signaling pathway.

**FIGURE 7:**
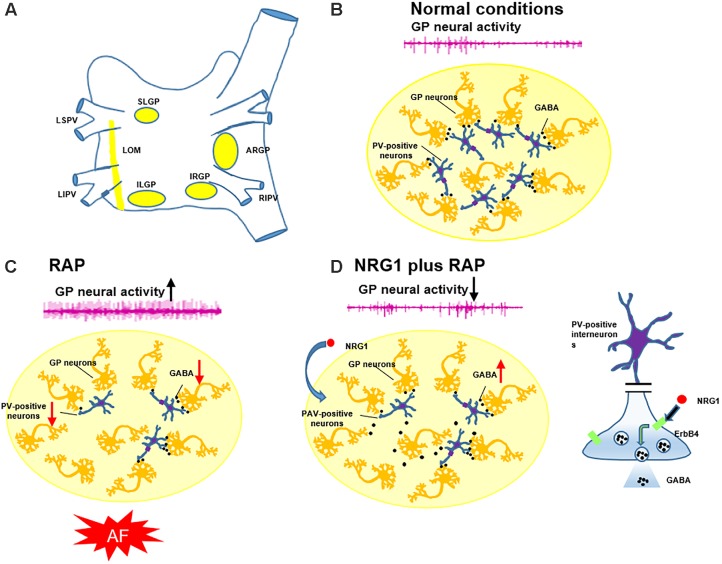
Schematic diagram illustrating the involvement of PAV-positive neurons in the genesis of AF. **(A)** The position of four major GP and LOM. **(B)** PAV-positive neurons (inhibitory neurons) play an important role in modulating the GP neural activity in normal conditions. **(C)** AF simulated by RAP suppressed PAV-positive neurons, diminished evoked GABA release, and increased GP neural activity and promoted AF. **(D)** Pretreatment with NRG1 activated PAV-positive neurons and enhanced evoked GABA release via ErbB4 receptors, which suppressed RAP-induced GP activation and AF.

Previous studies have indicated that NRG1/ErbB4 expression was restricted to GABAergic interneurons, and its expression was particularly high in PAV-positive neurons (Vullhorst et al., 2009; Wen et al., 2010). Suzukawa et al. (1999) found that the loss of perikaryal PAV immunoreactivity and overexpression of c-fos and NGF were involved in the development of epileptogenesis after ethacrynic acid-induced seizure. C-fos is a rapid indicator of neuronal activation, and it is broadly used as a marker for fast neuronal activation (Dragunow and Robertson, 1987). NGF is a potential novel biomarker of sympathetic neurons, and it is a neurotrophic factor that initiates pathological sympathetic neural growth in damaged cardiac tissue (Lazarovici et al., 2006). Previous studies have indicated that NGF was related to increasing sympathetic tone, and autonomic intervention may suppress AF via inhibition of NGF expression (Zhou et al., 2004). We observed that 6h-RAP induced more expression of c-fos and NGF protein and PAV-positive neurons in the GP (**Figure 6**). The PAV-positive neurons may modulate the intrinsic cardiac autonomic nervous system after atrial electrical remodeling (**Figure 7**).

It is well known that eNOS activation and vagal modulation play a role in paroxysmal AF (Fatini et al., 2006; Yu et al., 2013). Activation of the M2 muscarinic cholinergic receptor activates the isoform of nitric oxide synthase in endothelial cells of cardiac muscle (Feron et al., 1997), and ErbB4 signaling is critical for eNOS activation and the modulation of vagally mediated changes in NO production (Feron et al., 1999). Our investigation demonstrated a potential mechanism of the NRG1/ErbB4 signaling system in a paroxysmal AF model. Six-hour RAP induced acute atrial electrical remodeling and AF via activation of the intrinsic cardiac autonomic nervous system (Lu et al., 2008), and microinjection of NRG1 prevented activation of cardiac neural activity. The detailed mechanism by which ErbB4 and nitric oxide synthase function in paroxysmal AF is not known. More research is needed to further elucidate the detailed mechanism.

### Clinical Implications

Atrial fibrillation is the most common arrhythmia, and its prevalence is expected to increase sharply as the population ages (Shen et al., 2011). A significant portion of these patients will exhibit drug-refractory AF and require ablation (Benjamin et al., 1998). Catheter or surgical ablation carries significant risks of serious complications. The present study demonstrated that activation of the NRG1-ErbB4 signaling pathway in the atrial GP was a potential mechanism of GP hyperactivity and a contributing factor to AF initiation and maintenance. The NRG1-ErbB4 signaling pathway may be a new target for the activation of PAV-positive neurons via the NRG1/ErbB4 pathway and suppression of RAP-induced GP activation, atrial electrical remodeling and AF. Therefore, pharmacological regulation of the NRG1/ErbB4 pathway is a potential treatment for patients in an early stage of AF (e.g., paroxysmal AF).

### Study Limitation

Previous studies demonstrated that the atrial muscle played a role in the NRG1/ErbB4 signaling system (Feron et al., 1999; Zhao et al., 1999). The possibility that the shortening of ERP in response to RAP may be a protective response to limit calcium overload and M2 muscarinic cholinergic receptors activation of nitric oxide synthase in endothelial cells of cardiac muscle may be the physiological mechanism. Isolated atrial myocytes for patch and confocal image evaluations of the impact of NRG1 on myocyte electrophysiology are needed to verify this hypothesis in the future. We did not clearly identify PAV-positive neurons in the ARGP, and whether the other major atrial GP was affected in the same manner was not investigated. Further work must be undertaken to prove the existence of PAV-positive neurons within the cardiac ganglia and its importance and influence on the electrical conduction and propagation of cardiac impulses. Second, the neurochemical profile of the neurons within the intrinsic cardiac nervous system is complex, and we did not block NRG1-ErbB4 signaling by neutralizing NRG1 with ecto-ErbB4 to confirm the effect of NRG1 on GP. Third, we examined the effects of NRG1 on AF in acute canine models, and the long-term effect of NRG1 on AF is not clear. Further studies with long-term follow-up should be performed.

## Conclusion

Activation of the NRG1/ErbB4 pathway in atrial GP may be a potential mechanism in suppressing RAP-induced GP activation, atrial electrical remodeling and AF. Pharmacological regulation of the NRG1/ErbB4 pathway is a potential treatment for patients in an early stage of AF.

## Author Contributions

XZ, SP, and HJ contributed to the conception of the study. BH, SY, YW, and GM performed the experiments. XZ, ZW, BH, and XS contributed significantly to analysis and manuscript preparation. XZ, ZW, and BH performed the data analyses and wrote the manuscript. LY, SP, and HJ helped to perform the analysis with constructive discussions.

## Conflict of Interest Statement

The authors declare that the research was conducted in the absence of any commercial or financial relationships that could be construed as a potential conflict of interest.
